# Effect of* Caesalpinia coriaria* Fruits and Soybean Oil on Finishing Lamb Performance and Meat Characteristics

**DOI:** 10.1155/2018/9486258

**Published:** 2018-02-21

**Authors:** Nallely Sánchez, Germán David Mendoza, José Antonio Martínez, Pedro Abel Hernández, Luis Miguel Camacho Diaz, Hector Aarón Lee-Rangel, Anayeli Vazquez, Rogelio Flores Ramirez

**Affiliations:** ^1^Departamento de Producción Agrícola y Animal, Universidad Autónoma Metropolitana Xochimilco, Calzada del Hueso 1100, 04970 Ciudad de México, Mexico; ^2^Centro Universitario UAEM Amecameca, Universidad Autónoma del Estado de México, 56900 Toluca, MEX, Mexico; ^3^Facultad de Veterinaria, Universidad Autónoma de Guerrero, Chilpancingo, GRO, Mexico; ^4^Facultad de Agronomía y Veterinaria, Universidad Autónoma de San Luis Potosí, km. 14.5 Carr. San Luis Potosí-Matehuala, 78321 San Luis Potosí, SLP, Mexico; ^5^CIACYT, Universidad Autónoma de San Luis Potosí, 78000 San Luis Potosí, SLP, Mexico

## Abstract

To evaluate phenolic compounds and whether the combination of a tanniferous fruit and soybean oil could improve the performance, meat characteristics, and fatty acid (FA) profile in lambs, an experiment was conducted over 40 days with twenty creole male lambs (23.71 ± 3.46 kg). The lambs were allotted in a completely randomised design, with factorial arrangement 2 × 2, with the following dietary treatments: (1) control diet, (2) 2%* Caesalpinia coriaria* ground fruit dry matter (DM), (3) 2% soybean oil DM, and (4) 2%* Caesalpinia coriaria* fruit plus 2% soybean oil. The concentration of condensed tannins (CT) in* Caesalpinia coriacea* was 21.71 g/kg DM. No interactions were detected (*P* > 0.05) among soybean oil and* Caesalpinia coriaria*, and there were no differences in daily gain, intake, and feed conversion. Soybean oil reduced (*P* < 0.05) DM digestibility (68.05 versus 59.56%). In fat from the* longissimus thoracis et lumborum *(LTL) muscle, only linoleic acid presented differences (*P* < 0.05) between treatments. The combination of* Caesalpinia coriacea* fruit and soybean oil did not improve lamb performance at the included levels.

## 1. Introduction

There is interest in the study of local plants and to develop extracts that contain secondary compounds (condensed tannins and saponins) that, in addition to their potential to reduce methane emissions, have nutraceutical effects for ruminants [1] and many metabolites with antioxidant properties [2]. Besides it is possible that some phytochemicals promote the synthesis of conjugated linoleic acid, which would have beneficial effects on the consumer due to its nutraceutical effects (cardiovascular, anticancer, neuroprotective, antiosteoporotic, anti-inflammatory, and antioxidant) in humans [3].

The inclusion of plants with condensed tannins (CT) in low or moderate levels in ruminant diets has demonstrated beneficial effects on lamb performance because the reduction in ruminal protein degradation improves the amino acid supply to the small intestine [4, 5]. Tannins may also reduce losses of methane, and this could increase ME for growth;* in vitro* studies have shown that increasing graded levels of tannin-containing tropical tree leaves result in a linear reduction in methane concentration [6]. Other benefits from tannin supplementation in lamb rations are a higher deposition of Trans C18:1 and C18:2 n-6 in lamb muscle [7].

Vegetable oils are of interest in ruminant feeding since they can reduce methane emissions [8] but there are few evaluations in combination with plants rich in phytochemicals. In an experiment with feedlot lambs with increasing levels of a tanniferous bush and vegetable oils (soybean oil and linseed oil 2 : 1), there were no reports of interactions or effects on growth; even at levels of 80 g/kg of oil, intake was depressed. However, oil supplementation increased total n-3 polyunsaturated fatty acids in meat, improving nutritional value [9]. The inclusion of soybean oil in lamb finishing diets at 60 g/kg did not affect the feedlot performance of lambs [10]. Soybean oil has been reported to have an inhibitory effect on methane production when included at 30 g/kg, affecting methanogenic bacteria and rumen protozoa in lambs [11].

The effects of different tanniferous plants on polyunsaturated fatty acids (FA) in meat indicate that not all condensed tannins have the same effect on ruminal biohydrogenation [12] of dietary fatty acids (FA) as linoleic acid, which improves the rise of trans-11 18:1 (VA, vaccenic acid) in the rumen and thus the content in meat products. Therefore, different plants need to be evaluated to identify those that induce beneficial effects in lamb performance and meat quality. The leaves of* Caesalpinia coriaria* have a very rich tannin content with moderate antibacterial activity against pathogen bacteria [13], and some tropical fruit tree species contain tannins in substantial concentrations [14, 15]; therefore, it was hypothesised that the inclusion of dehydrated fruits with tannins in ruminant diets at lower levels may show the beneficial effects of condensed tannins. An experiment was designed to evaluate whether the inclusion of* Caesalpinia coriaria* fruit, with or without soybean oil, in feedlot rations could improve lamb performance, meat quality, and long-chain fatty acid deposition in muscle.

## 2. Materials and Methods

The Animal Care and Use Committee of the Doctorate Program in Animal and Agricultural Sciences from the Universidad Autónoma Metropolitana Campus Xochimilco approved the procedures.

Twenty male hair crossed lambs (23.71 ± 3.46 kg) were randomly assigned to one of four treatments (*n* = 5 lambs/treatment): (1) control diet, (2) 2%* Caesalpinia coriaria* dehydrated ground fruit DM, (3) 2% soybean oil DM, and (4) 2%* Caesalpinia coriaria* fruit and 2% soybean oil. The lambs were housed in individual crates, and feed was provided at 08:00 and 15:00 h. The lambs were adapted to the experimental diets (Table 1) for 10 days, and the experiment lasted 40 days. All lambs had free access to feed, ensuring 100 g orts per kg of the amount fed daily.

The fruits of* Caesalpinia coriaria* were collected in February 2014 in the Pungarabato municipality of Mexico. The fruits were mixed and dried in the shade for 15 days, and, then, the whole fruit (containing the peel and seed) was ground in a hammermill containing a screen size of 4 mm. Other feeds from the ration were ground with a 2.0 cm screen and mixed in a grinder-mixer (Vigusa, Mexico) to offer experimental rations as total mixed rations.

Daily samples of the feed and orts were collected and combined every 14 days. The dry matter and nitrogen in the diets were analysed according to the AOAC [16]. Neutral detergent fibre (NDF) and acid detergent fibre (ADF) analyses were conducted with a detergent system [17]. Fecal samples were collected every 4 days [18] up to day 30 of the experimental period to estimate apparent dry matter digestion. Feed and orts were collected daily during the same period. Acid-insoluble Ash was used on samples as an internal marker to estimate DM and NDF digestibility [19].

The evaluated variables were daily feed intake, average daily gain (ADG), feed conversion ratio,* longissimus thoracis et lumborum *(LTL) muscle area, and meat characteristics. The muscle area from* longissimus dorsi *was assessed one day before slaughter (day 39) by ultrasonography [20]. After slaughter, muscle samples (5 g) of the left side loin (*longissimus thoracis et lumborum* (LTL)) were collected and stored in a freezer (−20°C) until analyses were performed. To measure Warner-Bratzler shear force, 2.5 cm thick steaks were cooked at 70°C, using a model 1132 Instron Universal Testing Machine (Instron, Canton, MA) with a Warner-Bratzler attachment [21]. Color was measured 24 h after slaughter in fresh cuts of the loin samples using a Minolta CM-2006d spectrophotometer (Konica Minolta Holdings, Inc., Osaka, Japan). The lightness (*L*^*∗*^), redness (*a*^*∗*^), and yellowness (*b*^*∗*^) were recorded [22].

In the dehydrated fruit of* Caesalpinia coriaria*, total condensed tannins (TCTs) were assayed using the butanol-HCl method [23], with* Lysiloma acapulcensis *being used as an internal standard [24]. Analyses of the free (free-CT), protein-bound (PCT) and fibre-bound (FCT) condensed tannins were conducted according to the method of Porter et al. [25]. The purification was performed with Sephadex LH-20 [26, 27].

Lipids for fatty acid analysis were extracted from 500 mg of muscle and analysed in a sample obtained from the area between the 11th and 12th ribs (2.5 cm^2^) using 2 : 1 (vol/vol) chloroform-methanol [28]. A total of 10–20 mg of extracted lipid was derivatized using 1 : 4 (vol/vol) tetramethylguanidine and methanol [29]. Fatty acid profiles were determined by chromatography on a Supelco-2560, 100 m · 0.25 mm 0.20 mm column (Sigma Aldrich Canada, Oakville, ON, Canada) installed in a gas chromatograph (Agilent 6890, Agilent United States, Santa Clara, CA, USA) by flame ionization detection and splitless injection. Fatty acids from the muscle samples were identified by comparison with retention times of known standards (Sigma Aldrich Canada).

The results were analysed according to a completely randomised design with a 2 × 2 factorial arrangement, and the means were compared with the Tukey test [30].

## 3. Results and Discussion

The contents of total tannins, tannins bound as fractions, and condensed tannins in the fruit are presented in Table 2, showing a high proportion of those bound to fibre and low proportion of those bound to the protein fraction. Most of the studies with tanniferous plants have been focused on the leaves, so there are little data for fruits such as* Crescentia alata *and* Guazuma ulmifolia *[15], perhaps because the intake of fruits by small ruminants in the field is limited due to the large size and hardness of the fruit, which require that mature fruit be collected [16], dehydrated, and ground to be included in the diet.

Even though* Caesalpinia coriaria* leaves have been considered rich in tannin content [13–31], the total CT in the fruit is lower compared to that found in plants consumed by goats in dry tropics where values range from 78 g/kg to 174 g/kg DM [32]. The CT in the fruit* (Caesalpinia coriaria)* is lower than the mean of tropical plants with a high tannin content of 30 g/kg DM [33] but can be considered similar to* Lotus corniculatus*, a legume adapted to acid soils with 23 g/kg condensed tannins [34]. However, the CT (21.71 g/kg) is higher than other tree tropical fruits with concentrations reported of 11.9 and 12.0 g/kg CT [15].

The proportion of tannins bound to fibre or protein in* Caesalpinia coriaria* fruit is relatively low when compared to tropical plants consumed by goats [32]. It has been shown that the condensed tannins have antimethanogenic effects, but the biological importance of different fractions has not been fully elucidated. Plants containing both hydrolysable plus condensed tannins were more effective in reducing the total in vitro gas and methane production than those containing only hydrolysable tannins [35].

No interactions were detected among soybean oil and* Caesalpinia coriaria* fruit; the main effects are presented (Table 3). There were no differences in most of the lamb performance variables; however, soybean oil reduced (*P* < 0.05) NDF digestibility (68.05 versus 59.56% and tended (*P* = 0.15) to reduce DM digestibility 84.08 versus 81.21%). DM and NDF digestibility were highly correlated (*r* = 0.96; *P* < 0.0001). Most of the loin meat characteristics were not affected by treatments.

Several studies agree that lamb performance is not improved with soybean oil or with plants rich in condensed tannins. Abdalla et al. [33] found that the addition of soybean oil at 1.8% and 3% in the diet did not affect lamb performance. Dávila-Ramírez et al. [10] reported similar results in body weight gain, feed intake, and feed efficiency with 6% soybean oil. Others with 2% [36] or 3% [11] soybean did not find a response in lamb performance, even when methane emission was reduced in this last study. In one experiment, when* Crescentia alata* and* Guazuma ulmifolia* fruits were offered ad libitum, the intake of* Crescentia alata* was minimal (48 g/d) compared to the* Guazuma ulmifolia* fruit (686 g/d), which severely affected daily gain in lambs (81 versus 4 g/d) [14]. In other experiments with fixed leaf ratios of* Crescentia alata *and* Guazuma ulmifolia fruits* (15 and 30%), daily gain and feed conversion were not affected, and only intake was higher with 30% of* C. alata *[15].

The dietary concentration of CT in this experiment (0.42 g/kg DM) was lower compared to other studies; the experiment with lamb rations including fruits [15] with concentrations from 1.7 to 3.6 g/kg and daily gain was similar to the control diet, while, in other experiments with higher concentration silages (32 and 62 g/kg DM), the daily gain was reduced at the highest concentrations. The study [5] included* Glycyrrhiza glabra* leaves in lambs with a dietary concentration of 4 g/kg of DM and found a positive response only when PEG associated with the protein.

In another experiment, dietary levels of condensed tannins of 5, 10, and 20 g/kg DM dietary* Cistus ladanifer* combined with a vegetable oil blend (0, 4 and 8%) were evaluated; there were no changes in weight gain by condensed tannins, but oil intake was reduced [9], as observed in our experiment.

Soybean oil may affect protozoa and methanogens; Mao et al. [11] found that the protozoa population was reduced to approximately 52% and methanogens to 41% in lambs fed a diet with soybean oil. This is confirmed in other studies where animals fed a rumen-protected fat diet show larger numbers of protozoa than those supplemented with soybean oil [37].

The effects of substrates containing tannins on ruminal fermentation are desirable if they do not alter VFA concentration and decrease both ammonia N and methane production [6]. The beneficial effects of condensed tannins in moderate dietary concentrations (20–40 g/kg DM) are associated with the improvement of amino acid supply to the small intestine [4] or by the protein sparing effect [5].

In this experiment, only the yellowness was higher with* Caesalpinia coriaria* at 2% (*P* < 0.05) in the diet. This may be an indicator that tannins could protect against oxidation. However, in other studies, color and shear force values of meat were not affected by the inclusion of plants with condensed tannins [9] or by dietary soybean oil [9–38].  Przywitowski et al. [39] found that diets containing 0.76 and 7.84 g/kg of tannins have positive results on color parameters of turkey meat (*a*, *b* and *L*). Tannin-rich extract could create a darker color and decrease yellowness values in the meat of lambs Luciano et al. [40]. Likewise, Du et al. [41] observed some changes in redness for dietary sorghum with a high tannin content diet; the phenolic compounds such as tannins might improve the color stability of broiler meat during storage [39].

Regarding the changes in long-chain fatty acids, only linoleic acid showed significative differences (*P* < 0.05; Table 4). Several natural phytochemicals and antioxidants, primarily phenolic compounds such as tannins, are also known to exert modulatory effects on lipids in ruminants [42] by decreasing cholesterol levels and altering the FA profile of meat. Vasta et al. [7] reported the concentration of PUFA longissimus muscle from lambs fed the tannin-containing diets (4% condensed tannins). Similar to our results, Min et al. [43] observed changes in C18:2n-6 in growing goats fed with different levels of tannins, but they increased 18:1 n-7c, 18:1 n-7t, 18:2 n-6c, 18:3 n-3, and 20:2 n-6 compared to the control diet.

Possibly, the higher secretion of C18:1n9t and 18:1n9c with soybean oil could be due to partial biohydrogenation of dietary cis-9 C18:1 to C18:0 followed by its desaturation by stearoyl-CoA desaturase and due to partial protection of soybean oil cis-9 C18:1 from ruminal biohydrogenation [44]. Tannin-containing diets reduce ruminal biohydrogenation. This implies that tannin supplementation could be a useful strategy to increase rumenic acid and polyunsaturated fatty acid concentration and decrease the saturated fatty acids in lamb meat [43].

## 4. Conclusion

The inclusion of 2%* Caesalpinia coriaria* fruits, 2% soybean oil dry matter, or their combination does not improve lamb performance in finishing lamb rations, although it could affect long-chain fatty acids in lamb meat.

## Figures and Tables

**Table 1:** Experimental diets and chemical composition.

	Control	*Caesalpinia coriaria*	Soybean oil	*C. coriaria* + soybean oil
Ingredients (as fed basis, %)				
Sorghum grain	19.608	19.608	19.608	19.231
Corn grain	34.804	34.804	34.804	34.135
Soybean meal 46% CP	3.922	3.922	3.922	3.846
Molasses sugar cane	11.765	9.804	9.804	9.615
Rice polish	8.824	8.824	8.824	8.654
Soybean hulls	5.882	5.882	5.882	5.769
Urea	0.980	0.980	0.980	0.962
Canola meal	3.431	3.431	3.431	3.365
Corn gluten meal	1.961	1.961	1.961	1.923
Wheat bran	3.922	3.922	3.922	3.846
Calcium carbonate	1.471	1.471	1.471	1.442
Mineral premix^1^	0.980	0.980	0.980	0.962
Sodium bicarbonate	0.980	0.980	0.980	0.962
Sodium chloride	0.490	0.490	0.490	0.481
Inert fat	0.980	0.941	0.941	0.808
*Caesalpinia coriaria *	-	2.000	-	2.000
Soybean oil	-	-	2.000	2.000
Total	100.000	100.000	100.000	100.000
Nutrient content				
DM%	94.910	94.750	94.690	94.530
Ash%	8.990	9.460	8.900	9.370
Ether extract%	3.940	5.675	3.241	5.577
Crude protein%	14.604	13.961	14.327	13.944
NDF%	29.739	30.150	28.953	30.489
ADF%	14.665	15.255	14.319	15.252

^1^NaCl 3000 g, Co 75 mg, Cu 5,000 mg, Cr 200 ppb, P 40 g, Fe 30,000 mg, Mn 2,000 mg, Se 100 mg, I 125 mg, Zn 10,500 mg, vitamin A 6,800,000 IU, vitamin D 630,000 IU, and vitamin E 16,500 IU.

**Table 2:** Tannin content in *Caesalpinia coriacea* fruit.

Condensed tannins g/kg	21.71
Bounded to protein g/kg	3.17
Bounded to fibre g/kg	7.18
Total tannins g/kg	32.06

**Table 3:** Main effects of *Caesalpinia coriaria* fruit and soybean oil on lamb performance and digestibility.

	Control	*Caesalpinia coriaria*	Soybean oil	*C. coriaria* + soybean oil	SEM
Initial BW kg	23.95	23.48	23.46	23.97	1.777
Final BW kg	35.64	35.53	34.74	36.43	1.490
DM intake kg/d	1.254	1.300	1.249	1.305	0.035
ADG kg	0.292	0.301	0.282	0.311	0.016
Feed conversion	4.44	4.42	4.59	4.26	0.252
DM digestibility%	84.08	81.21	83.36	81.93	2.180
NDF digestibility%	68.05^a^	59.56^b^	66.09^a^	61.52^b^	0.775
Ruminal pH	5.05	5.64	5.67	5.02	0.407
LTL area cm^2^	841.1	980.6	957.0	864.7	75.46
Color characteristics					
*L*	37.02	36.74	36.07	37.69	0.548
*a*	20.78	19.63	20.45	19.96	0.616
*b*	6.96^ab^	6.64^ab^	5.86^b^	7.73^a^	0.456
WBSF kg/cm^2^	785.58	687.94	783.94	689.58	80.11

^a,b,ab^Means with different superscript within main effect are different (*P* < 0.05); *L*, lightness; *a*, redness; *b*, yellowness; WBSF: Warner-Bratzler shear force.

**Table 4:** Main effects of *Caesalpinia coriaria* fruit and soybean oil on fatty acids composition of muscle lipids.

	Control	*Caesalpinia coriaria*	Soybean oil	*C. coriaria* + soybean oil	SEM
C14:0	3.78	4.14	4.14	3.64	0.94
C14:1-9	0.14	0.28	0.75	0.23	0.12
C16:0	29.17	25.08	30.15	28.73	4.21
C16:1	3.69	5.05	4.44	3.26	1.43
C18:0	21.44	22.39	15.88	17.84	5.25
C18:1	0.24	0.33	0.46	0.42	0.11
C18:2n-6	4.12^ab^	0.46^b^	4.67^a^	2.35^ab^	0.82
C18:3n	0.15	0.11	0.25	0.15	0.55

^a,b,ab^Means with different superscript within main effect are different (*P* < 0.05).
